# Exome sequencing and targeted gene panels: a simulated comparison of diagnostic yield using data from 158 patients with rare diseases

**DOI:** 10.1590/1678-4685-GMB-2021-0061

**Published:** 2021-09-29

**Authors:** Caio Robledo D’Angioli Costa Quaio, María José Rivadeneira Obando, Sandro Felix Perazzio, Aurelio Pimenta Dutra, Christine Hsiaoyun Chung, Caroline Monaco Moreira, Gil Monteiro Novo, Patricia Rossi Sacramento-Bobotis, Michele Groenner Penna, Rafaela Rogerio Floriano de Souza, Vivian Pedigone Cintra, Juliana Emilia Prior Carnavalli, Rafael Alves da Silva, Monize Nakamoto Provisor Santos, Daniele Paixão, Wagner Antonio da Rosa Baratela, Caroline Olivati, Gustavo Marquezani Spolador, Maria Carolina Pintao, Alexandre Ricardo dos Santos Fornari, Matheus Burger, Rodrigo Fernandes Ramalho, Otavio Jose Eulalio Pereira, Elisa Napolitano e Ferreira, Miguel Mitne-Neto, Chong Ae Kim

**Affiliations:** 1 Universidade de São Paulo, Faculdade de Medicina, Hospital das Clínicas (HCFMUSP), Instituto da Criança, São Paulo, SP, Brazil.; 2 Fleury Medicina e Saúde, São Paulo, SP, Brazil.; 3 Hospital Israelita Albert Einstein, Laboratório Clínico, São Paulo, SP, Brazil.; 4 Universidade Federal de São Paulo, Divisão de Reumatologia, São Paulo, SP, Brazil.; 5 Universidade Federal de São Paulo, Escola Paulista de Medicina, Laboratório de Hepatologia Molecular Aplicada (LHeMA), São Paulo, SP, Brazil.

**Keywords:** Exome sequencing, Next-generation sequencing, targeted gene panels, diagnostic yield, NGS panel

## Abstract

Next-generation sequencing (NGS) has altered clinical genetic testing by widening the access to molecular diagnosis of genetically determined rare diseases. However, physicians may face difficulties selecting the best diagnostic approach. Our goal is to estimate the rate of possible molecular diagnoses missed by different targeted gene panels using data from a cohort of patients with rare genetic diseases diagnosed with exome sequencing (ES). For this purpose, we simulated a comparison between different targeted gene panels and ES: the list of genes harboring clinically relevant variants from 158 patients was used to estimate the theoretical rate of diagnoses missed by NGS panels from 53 different NGS panels from eight different laboratories. Panels presented a mean rate of missed diagnoses of 64% (range 14%-100%) compared to ES, representing an average predicted sensitivity of 36%. Metabolic abnormalities represented the group with highest mean of missed diagnoses (86%), while seizure represented the group with lowest mean (46%). Focused gene panels are restricted in covering select sets of genes implicated in specific diseases and they may miss molecular diagnoses of rare diseases compared to ES. However, their role in genetic diagnosis remains important especially for well-known genetic diseases with established genetic locus heterogeneity.

## Introduction

Next-generation sequencing (NGS) has altered dramatically clinical genetic testing by reducing the cost and widening the access to molecular diagnosis of rare diseases with genetic etiology. Genomic capture is a step in the NGS methodology that can target specific areas in the genome, including subsets of genes of interest or even all known human genes, but this limits the study to what is already known in one hand and reduces the cost on the other (Teer and Mullikin, 2010). As there are numerous molecular tests available, including exome sequencing (ES) and vast amounts of gene panels, physicians may face difficulties selecting the best diagnostic approach for their patients with suspected genetic conditions.

Focused gene panels are restricted in covering select sets of genes implicated in specific diseases. Therefore, they demand some sort of prior clinical diagnostic hypotheses driven by clinical examination and previous exams, which is difficult in several instances when dealing with rare diseases, especially when they present atypical presentations or clinical overlap with other conditions. Clinical laboratories have been providing this genetic diagnostic approach for more than a decade, though laboratories usually show different strategies in selecting the sets of genes for their panels. This makes the lists of genes, thus the sensitivity of the panels, vary widely between different panels from different labs and, sometimes, different panels within the same lab. Another remaining challenge for gene panels is that, given the vast number of genes potentially involved in atypical presentations of rare disorders, and our fast growing understanding of rare diseases, choosing a gene panel which is both specific enough to detect the cause of the suspected genetic disease and broad enough to include all known genes associated with the condition and all differential diagnoses is often a challenging task (Xue et al., 2015). On the other hand, there are advantages in choosing a focused panel: greater coverage of targeted regions and higher depth per base, relatively easier interpretation of the report results and avoiding secondary findings, which are occult deleterious genetic variants associated with clinically relevant conditions besides the primary objective of analysis (Klitzman et al., 2013).

ES can be considered a more unbiased diagnostic method for monogenic diseases because it allows a much broader net for molecular diagnoses. In this method, the great majority of known protein-coding human genes are captured. Although this approach is not limited to specific sets of genes and theoretically includes the great majority of human genes, it still relies on clinical data for the interpretation of genomic information (Xue et al., 2015).

In this article, we simulate a comparison to estimate diagnostic yields of targeted gene panels from different laboratories using data from a cohort of patients with rare genetic diseases diagnosed with ES. For this purpose, we tested whether our patients diagnosed with ES would have been diagnosed by NGS panels by checking if the gene that harbored pathogenic variants was part of the list of genes of the respective panel.

## Material and Methods

We reviewed the clinical indications and primary findings of ES analysis from our cohort of 500 symptomatic patients who had undergone molecular investigation for suspected diseases of genetic etiology from 2016 to 2020 in facilities of the Fleury Group. All patients or their legal guardians provided consent before exome analysis and this study was granted ethics committee approval from both institutions involved (Plataforma Brasil; CAAE# 02617018.3.0000.5474; Fleury# 3.372.339).

### Selection of patients, molecular analysis, bioinformatics protocols

A total of 500 patients had been referred for exome sequencing in our original cohort (cases #1 through #500; Quaio et al., 2020, 2021). All the samples were collected from 2016 to 2020 in facilities of the Fleury Group, a tertiary private general diagnostic laboratory with subsidiaries in nine states of Brazil and based in São Paulo, Brazil. This cohort is restricted to symptomatic patients who had undergone molecular investigation for suspected diseases of genetic etiology. All patients or their legal guardians provided consent before exome analysis. Patients who refused to share genomic data or receive information on medically actionable findings were excluded from this cohort. Additionally, exome analysis performed for other reasons than clinical diagnostic (such as research protocols or prenatal genetic counseling) were excluded from our review. Thirty-one cases that did not meet these inclusion criteria were excluded.

DNA from the proband and both parents were extracted in a clinical setting from peripheral blood leukocytes or saliva. Exome capture was also conducted in a clinical setting using Agilent Clinical Research Exome v1 according to the manufacturer’s instructions. Sequencing was performed using an Illumina NextSeq platform. Exome data were aligned to the GRCh37.75/hg19 reference genome using the Burrows-Wheeler Aligner (BWA; version 0.7.17-r1188). Variants (single-nucleotide variants [SNVs] and indels) were identified following the best practices of the Broad Institute using the Genome Analysis ToolKit (GATK, version 3.8-0-ge9d806836) software and annotated using Variant Effect Predictor (VEP, version 88.14). All the exomes met a minimum of 95% of target bases covered at >10×. In-house bioinformatic pipelines were developed by a dedicated team of specialists. The mitochondrial genome and copy number variants were not studied.

### Variant classification, clinical data, and molecular findings

At least two independent NGS analysts were responsible to preselect variants considering an allele frequency <1%, variant functional impact, clinical relevance of gene harboring the variant, relevant reports from databases (e.g., ClinVar and HGMD) and the literature. These preselected variants were then discussed in a board comprising three experts. After this first board meeting, selected variants would undergo through confirmation and segregation studies by Sanger sequencing when the parental samples were available. Paternity and maternity were not confirmed by any specific test in our cohort, although nonpaternity/nonmaternity could be disclosed by genotyping multiple rare variants. Variants were classified according to ACMG guidelines with assistance of a third-party ACMG calculator by Saphetor SA (Lausanne, Switzerland) (www.varsome.com).

The clinical data were collected through a comprehensive pretest form completed by the attending physician or family, medical reports and clinical notes provided to the laboratory. Patients were not evaluated by specialists from our team. For the purpose of this article, the clinical features provided to the laboratory were divided as follows: 1) Neurodevelopmental disorders, 2) Seizure, 3) Syndromic/malformative, 4) Immune/hematological diseases, 5) Cardiovascular disease and 6) Metabolic abnormality. One patient may be part of different groups, depending on his/her clinical manifestations. This clinical division was used to select NGS panels, as stated below. Clinical details of all patients are available in Table S1 .

Primary findings were reported in 158 patients. We defined as primary findings the variants considered as clinically relevant, observed in a gene that was associated with the patient’s phenotype, with compatible zygosity and an adequate inheritance pattern; these cases were considered positive. The other 342 patients had a negative exome analysis.

Considering all 158 patients to whom primary findings were reported, we have the following distribution among clinical groups: Neurodevelopmental disorders (n=82), Seizure (N=36), Syndromic/malformative (n=72), Immune/hematological diseases (n=18), Cardiovascular disease (n=16) and Metabolic abnormality (n=17). One patient may be part of different groups. This clinical division was used to select NGS panels, as stated below. Clinical details of all patients are available in Table S1.

ES was considered in this work as the gold-standard for molecular diagnosis of monogenic, rare diseases because it is the most comprehensive genetic exam clinically available in Brazil nowadays. The list of genes harboring clinically relevant variants of the 158 patients from our cohort of rare diseases that underwent ES was used to estimate the theoretical rate of diagnoses missed by NGS panels from different laboratories.

Our team selected eight genetic clinical laboratories based on their national and international recognition of excellence, comprehensiveness of portfolios and availability in Brazil. Five of them are national laboratories, among which one is a public institution and the others are private providers. The other three are international laboratories: two from the United States and one from Germany. The panels and respective gene lists were selected according to the clinical indication stated above and information publicly available in laboratory websites and direct contact by email or call center. The names of the laboratories will not be disclosed because the objective of our study is not the comparison between different services, but the comparison of diagnostic methods: exome x targeted gene panels.

We selected 53 different NGS panels from eight different laboratories to simulate the estimated rate of possible missed diagnoses based on the list of genes available studied by these panels. Commercial provider websites were accessed between October and November 2020 to obtain gene coverage. The panels were required to sufficiently cover the differential diagnoses provided by clinical notes; if there were more than one commercial option available for each panel, we tried to identify the most comprehensive options considering gene lists. In some cases, we identified the availability of two options for the same laboratory and both options were included in the analysis. Gene coverage details for every panel and for each patient are available in Tables S2-S7. We took the conservative approach and assumed that the analytical sensitivity and specificity of all panels were 100% based on a previous study (Dillon et al., 2018).

Unpaired Student’s *t* test was used to determine significantly different means.

## Results

Our cohort originally consisted of 500 symptomatic patients who had undergone ES for diagnostic purpose. At least one molecular diagnosis of a genetic disorder was reported for 31.6% (n=158). These 158 cases with positive findings were used to simulate the rate of estimated diagnoses missed by NGS panels by calculating how many of them would not have had a definitive molecular diagnosis if they had been investigated by NGS panels instead of ES. Individual data for each case (brief case overview) are available in Table S1.

The estimation of missed diagnosis per Clinical manifestation and also per laboratory is summarized in Table 1. Overall, the mean rate of missed molecular diagnoses considering all 53 panels was 64%, varying from 14% (Lab H, Seizure panel; panel predicted sensitivity of 86%) to 100% (Lab A, Immune/hematological diseases panel; panel predicted sensitivity of 0%). Metabolic abnormalities represent the group of conditions with highest mean of missed diagnoses (86%; predicted average sensitivity of 14%), while seizure represents the group with lowest mean (46%; predicted average sensitivity of 54%).

**Table 1 -:** Rate of molecular diagnoses missed by NGS panels from the eight laboratories per clinical manifestation. In the first column, “n” represents the number of patients in the group; Labs A through E are Brazilian national laboratories, while F through H are international; column “Mean” represents the mean of missed diagnoses per group of Clinical Manifestation.

Clinical manifestation	Number of panels	Rate of missed diagnoses of NGS panels by Laboratory									
		Lab A	Lab B	Lab C	Lab D	Lab E	Lab F	Lab G	Lab H	Mean rate of missed diagnoses	Predicted average sensibility
Neurodevelopmental disorders (n=54)	9	74%	N/A	69%	22-87%	65%	22-39%	87%	48%	57%	43%
Seizure (n=36)	11	58-61%	64%	42%	25%	25-83%	47%	42%	14-47%	46%	54%
Syndromic/malformative (n=72)	4	N/A	63%	61%	60%	N/A	53%	N/A	N/A	59%	41%
Immune/hematological diseases (n=18)	9	89-100%	28%	28-78%	N/A	22%	33%	33%	39%	50%	50%
Cardiovascular disease (n=16)	10	81-88%	88%	63%	88%	69%	69%	88%	69-75%	78%	22%
Metabolic anomaly (n=17)	10	94%	94%	88%	59%	94%	53-88%	82-94%	94%	84%	16%

Based on each laboratory individually, the mean of missed diagnoses considering all panels per laboratory varied from 51% (predicted laboratory sensitivity of 49%) to 81% (predicted laboratory sensitivity of 19%). Panels from Brazilian laboratories missed more diagnoses (68%, corresponding to a sensitivity of 32%) compared to international laboratories (58%, corresponding to a sensitivity of 42%); this difference showed statistical significance (Unpaired t test, p<0.0001; 95% confidence interval of this difference: from 9.29933 to 10.70067).

We have estimated that the rate of missed molecular diagnosis of 158 patients with monogenic diseases would be on average 64% if they had been studied by NGS panels instead of ES, which represents a sensitivity of 36%. This rate ranged widely from 14% to 100%, depending on the clinical indication for testing and the laboratory where the test was performed.

The rate of missed diagnoses varied not only within different labs, but also when comparing different clinical indications and surprisingly different panels for the same diseases within the same laboratory. The biggest variation within the same lab comprises two different panels for Seizure in Lab E, with missed diagnoses rates of 25% and 83%. Patients with metabolic abnormalities and cardiovascular diseases were estimated with rates of missed diagnoses above 70%, which represented the highest rates, while the only group with missed diagnoses rate less than 50% corresponded to patients with seizure. Based on these rates, we have estimated the diagnostic yield of NGS panels, shown in Table 2.

**Table 2 -:** Diagnostic yield of exome sequencing in our cohort of 500 patients with rare diseases (Quaio CRDC et al., 2020) and projected diagnostic yield of NGS panels per clinical indication in the same cohort considering the rates of missed molecular diagnoses shown in Table 1.

Clinical manifestation	Diagnostic yield	
	Exome sequencing	Projected yield for NGS panels
Neurodevelopmental disorders	35%	15%
Seizure	34%	18%
Syndromic/malformative	41%	17%
Immune/hematological diseases	19%	10%
Cardiovascular disease	31%	7%
Metabolic anomaly	33%	5%

## Discussion

We used data from a cohort of patients with rare genetic diseases diagnosed with ES to estimate possible molecular diagnoses missed by different NGS panels from different laboratories and observed that targeted gene panels might miss an average of 64% of the diagnoses of our cohort. Although these high rates of missed diagnoses are remarkable, our methodology presents several limitations that does not allow to a definite conclusion towards the absolute advantage of exome sequencing over targeted gene panels, as we will discuss below.

Literature regarding comparison of NGS diagnostic methods is limited in Latin America and in other populations outside North America and Europe. As we did not find comprehensive studies that address this subject in Brazil or any other Latin American countries, we hope that our data may enrich this discussion and encourage further studies from other groups.

Our objective was to simulate the proportion of ES-diagnosed patients that would not have been covered by targeted gene panels offered by commercial-testing laboratories available in Brazil. For this purpose, we selected 53 different targeted gene panels from eight different clinical laboratories. We observed that the selection of genes for similar clinical indications varied significantly among panels from different clinical laboratories, possibly widening the range of missed diagnosis rates for each laboratory individually. These differences in the number of genes and also in the types of genes included in each panel rely basically on the criteria of inclusion that may reflect not only evidence from the literature, but also commercial purposes. The discrepancies between the gene panels might also be a result of a lack of consensus in the process of determining the genes clinically relevant to be included in the test as the inclusion criteria may be somewhat subjective.

Another important issue regarding targeted gene panels is that they may become outdated very quickly; some studies have demonstrated that an important number of genetic diagnoses encompass newly discovered diseases: around one fourth are within genes characterized within the last two years and 7% are novel gene discoveries (Farwell et al., 2015; LaDuca et al., 2017). Additionally, another important advantage of ES is the possibility of regular reanalysis of negative cases, which has been demonstrating as beneficial in the individual, familial and health system perspectives and improve diagnostic rates over time (Fung et al., 2020). Considering these data, we expect that targeted gene panels dating more than two years are more likely to miss an average of almost one third of molecular diagnoses compared to ES.

Several laboratories offer different panels for the same clinical purpose, which is an important limitation not only to the selection of panels that we have made in this study, but also for the clinicians in the clinical setting. Choosing the most appropriate set of genes is sometimes a challenge, as we will try to demonstrate through examples faced in this research.

Patient 126 was referred for the investigation of suspected metabolic disease because he presented weakness and hepatosplenomegaly. The clinical report also mentioned delayed tooth eruption, but it did not mention any details about myopathy or skeletal anomalies. Based on the clinical description provided, this case was attributed to the clinical group “Metabolic abnormality” and correspondent panels were to investigate whether this diagnosed would be missed. This patient was found with a dual molecular diagnosis (*DYSF*-related muscular dystrophy and *PTH1R*-related tooth agenesis) genes that were not included in any metabolic panel considered for this study. In this case, we believe that the clinical evaluation prior to molecular exam may have contributed for a negative targeted panel as gene panels are usually designed including genes associated with a specific phenotype spectrum and, in this example, our patient showed clinical features from two different phenotypic spectrum.

Here are some other examples of restricted clinical information provided to the laboratory: case 95 (clinical report mentioned hypoglycemia and clinical hypothesis of inborn error of metabolism), 211 (report of hypoglycemia and hepatomegaly) and 424 (report of hepatomegaly, hypoglycemia and jaundice: clinical suspicion of glycogen storage disease). These three cases were diagnosed as mitochondriopathies by ES (respectively *MPV17*, *POLG* and *MPV17*-related mitochondrial dysfunctions) and, probably would have been missed by most of the targeted gene panels studied here because several laboratories did not include mitochondrial genes in their most comprehensive metabolic panels.

These examples above raise an important factor that does not rely on the quality of gene selection criteria, but the importance of a comprehensive clinical evaluation in order to select the most sui genetic testing approach in order to confirm the clinical diagnosis. Some key factor may compromise this pre-test step for targeted gene panels: 1) several ordering physicians are not specialists in rare diseases and may lack a thorough knowledge in this field of expertise; 2) atypical manifestations of rare diseases may skew clinical evaluation towards a false clinical diagnosis and, therefore, an inadequate selection of gene panel.

Many studies have reported on the clinical utility of epilepsy gene panel testing, although they may present substantial variability in their diagnostic rates, ranging from as low as 10% to as high as 50% (Mercimek-Mahmutoglu et al., 2015; Butler et al., 2017; Rim et al., 2018; Jang et al., 2019). We have predicted diagnostic rate for epilepsy panels to be an average of 18%, ranging from 5.8% (Laboratory E) to 29.2% (Laboratory H).

Some studies have also compared ES to targeted gene panels for neurodevelopmental disorders. One study compared gene panels from 21 laboratories and observed that the number of genes ranged from 11 to 2,562 and ES diagnoses were made in genes not included in at least one commercial panel in 42% of cases (Dillon et al., 2018). In our study, we have predicted a mean rate of missed diagnoses slightly higher (57%), ranging from 22% (Laboratories D and F) to 87% (Laboratories D and G).

Our evaluation of metabolic diseases panels presents the most discrepant estimative for diagnostic rate. While we have estimated an average sensibility of 15% (ranging from 6% [Laboratories A, B, E, G, H] to 44% [Laboratory F]), studies have shown discrepant results varying widely, from 9.8% up to 50% (Ghosh et al., 2017; Lee et al., 2020). Some studies, though, have obtained a low diagnostic rate (around 15%) for cases with non-specific markers for metabolic diseases (Yubero et al., 2016). This is probably one of the most challenging group of diseases, because it encompasses hundreds of different clinical entities and several factors may interfere in diagnostic rates, including symptom onset age, gender, clinical course, organ systems involved, developmental history, family history and prior genetic testing (Lee *et al*., 2020).

Although ES offers a much broader range of genes compared to any panel, and studies have found that ES can detect more than 98% of pathogenic variants identified on different subset of gene panels (LaDuca et al., 2017), it is important to highlight that for several well-known genetic diseases with established genetic locus heterogeneity for the great amount of cases, a gene panel approach remains appropriate for an efficient and timely molecular diagnosis (Xue et al., 2015). In fact, targeted gene panels may offer advantages over ES in some instances, such as reduced cost, shorter turn-around time, lack of secondary findings and higher coverage of targeted genes. Therefore, more genes are not necessarily better. We believe that two main clinical categories may have more benefits from targeted gene panels compared to exome sequencing: cancer susceptibility genes, to which panels generally present more efficient enrichment allowing the study of recurring atypical, noncoding molecular mechanisms (e.g., recurring known alterations in promoter or intronic regions), and X-linked intellectual disability, in which ES presents worse coverage compared to targeted panels (LaDuca *et al*., 2017).

Even though ES can bypass the need of a previous selection of genes driven by clinical diagnostic hypotheses, variant interpretation still remains dependent on clinical data. When clinical information of the patient is incomplete, the analysis and interpretation of ES might be more challenging when compared to panels due to the greater amount of data obtained. Another important issue to consider in ES is that this diagnostic method may unintentionally unravel occult deleterious genetic variants not associated with the primary objective of analysis, although associated with clinically actionable conditions, defined as “incidental” or “secondary findings” (Klitzman et al., 2013). On one hand, secondary findings may impact on individuals’ psychological health negatively by unfolding previously unknown serious risks of future life-threatening events, on the other hand they may positively impact life expectancy (Quaio et al., 2020).

Our work presents a strong selection bias because our cohort consists mostly of patients with nonspecific clinical manifestations. It is not difficult to suppose that patients with clinical manifestations highly specific for known clinical conditions caused by a restricted subset of genes tend to undergo investigation through NGS panels. This fact may have substantially underrepresented this group of genetic diseases in our cohort, thus overestimating the rate of missed diagnosis by NGS panels.

Another limitation is that gene selection of targeted gene panels is dynamic and changes over time. As our work is based on the October/November 2020 versions of gene panels, our results may overestimate the rate of missed diagnosis of more up-to-date panels; on the other hand, we may underestimate the rate of missed diagnosis of patients that underwent older versions of gene panels, especially if we consider that NGS panels may become outdated rather quickly (LaDuca et al., 2017).

Information about other genetic investigations (e.g., NGS panel) prior to ES was not available. Additionally, several cases lacked thorough phenotyping and the clinical features relied solely on information provided to the laboratory, restricting the quality and quantity of phenotype information available for clinical-molecular correlation at the time of interpretation. The inclusion criteria for clinical cases were broad and may have included patients without a proper referral for ES, particularly considering that not all the patients were evaluated by clinical geneticists, some were referred by general practitioners, and other specialists without any training/experience in clinical genetics or dysmorphology.

The patients submitted to ES that remained without a molecular diagnosis continue to be a big challenge, especially if we consider that they comprised most of the patients (68.4%) from our original cohort with 500 individuals. A feasible, ongoing approach that is possible for patients submitted to ES with good cost-effectiveness is regular re-evaluation of ES data of nonpositive results with updated databases that include newly discovered disease-associated genes (Alfares et al., 2018). Another rising possibility is to study more complex interaction of rare variants in digenic or oligogenic predictors by studying combinations in gene pairs or networks, considering that some rare diseases may present digenic or even oligogenic inheritances (Renaux et al., 2019). Both approaches are more difficult and more limited using restricted data from panels compared to ES.

In conclusion, focused gene panels are restricted in covering select sets of genes implicated in specific diseases and they may miss an average of 64% of diagnoses compared to ES for cases of rare diseases without a clear clinical manifestation, though this rate varies widely depending on the clinical indication and laboratory. However, their role in genetic diagnosis remains important especially for well-known genetic diseases with established genetic locus heterogeneity because they still present some advantages, such as reduced cost, shorter turn-around time, and higher coverage of targeted genes.

## Supplementary material

The following online material is available for this article:

Table S1 ‒ Overview of patients.

Table S2 ‒ Neurodevelopmental disorders panels.

Table S3 - Seizure panels.

Table S4 ‒ Syndromic/malformative panels.

Table S5 ‒ Immune/hematological diseases panels.

Table S6 ‒ Cardiovascular disease panels.

Table S7 - Metabolic abnormality panels.
