# Supplementary material for: Exome sequencing and targeted gene panels: a simulated comparison of diagnostic yield using data from 158 patients with rare diseases
Source: Genet Mol Biol. 2021 Sep 29;44(4):20210061. doi: 10.1590/1678-4685-GMB-2021-0061 (PMC8485181; doi:10.1590/1678-4685-GMB-2021-0061)
Supplement: Table S1 ‒ [file 1415-4757-GMB-44-4-e20210061-s1.pdf]

Genetics and Molecular Biology

DOI: https://doi.org/10.1590/1678-4685-GMB-2021-0061

Supplementary Material to "Exome sequencing and targeted gene panels: a simulated comparison of diagnostic yield using data from 158 patients with rare diseases"

Table S1 – Overview of patients.

Summary of 500 Patients Investigated with Whole Exome Sequencing and with a positive diagnosis. Age in years. Abbreviations: M, male; F, female; het, heterozygous; hom, homozygous; hem, hemizygous; dn, *de novo*; var, variant.

Case ID	Gender	Neurodevelopmental disorders	Seizure	Syndromic/malformative	Immune/hematological diseases	Cardiovascular disease	Metabolic/hormonal anomaly	Primary Finding overview: Gene (zygosity, inheritance)
4	M	X		X				SLC52A2(hom)
6	M		X	X				NFIX(het, dn)
8	M			X				CSNK2A1(het, dn)
18	F	X	X					SLC13A5(hom)
22	F	X	X	X				MECP2(het, dn)
26	M	X		X				PQBP1(hem, inherited)
27	F	X		X				ARID1B(het, dn)
28	F	X		X				ZC4H2(het)
33	M	X	X	X				UPF3B(hem, inherited)
35	M							CLCN1(hom)
37	M	X		X			X	FAM111A(het, dn)
38	M	X						DYNC1H1(het, dn)
41	F	X		X				BLM(hom)
44	M	X	X	X				STXBP1(het, dn)
55	F					X		TNNC1(het, dn)
61	F							SETX(hom)
64	F							ABCB4(2 var in trans)
77	F						X	TF(hom)
78	F	X						MECP2(het, dn)
82	F	X					X	ITPR1(het, dn)
83	F							IGHMBP2(2 var in trans)
84	M	X	X	X				GNAO1(het, dn)
87	F	X	X					SLC2A1(het, dn)
94	F							AKR1D1(hom)

Case ID	Gender	Neurodevelopmental disorders	Seizure	Syndromic/malformative	Immune/hematological diseases	Cardiovascular disease	Metabolic/hormonal anomaly	Primary Finding overview: Gene (zygosity, inheritance)
95	M						X	MPV17(2 var in trans)
99	M	X	X				X	DEAF1(het, dn)
106	M							G6PC(2 var het, fase unknown)
108	M			X		X		ASXL1(het, dn)
109	F							NKX2-1(het)
110	F	X		X				PHF6(het, dn)
111	M							USH2A(2 var het, fase unknown)
114	F							SLC2A1(het, dn)
117	M	X						POLG(2 var in cis)
119	M	X		X				TGFBR2(het, inherited)
120	F	X	X				X	POLG(2 var in cis)
121	M	X	X	X				MECP2(hem, inherited)
124	F	X	X	X				GRIN2A(het, dn)
126	M						X	DYSF(2 var in trans), PTH1R(het, inherited)
127	F			X		X		LZTR1(het, dn)
129	F			X				FBN1(het, dn)
133	M	X						MYO7A(2 var in trans)
134	M					X		NDUFV1(2 var in trans)
136	F	X		X		X		MECP2(het, dn)
138	M	X						RNASEH2B(hom)
139	M	X		X				ITGA8(2 var in trans), PHF8(hem, inherited)
140	F	X		X				MECP2(het, dn)
146	M							RYR1(het)
148	M	X	X					ATP1A3(het, dn)
150	F							PHKG2(2 var in trans)
155	F			X				COL6A1(het, dn)
161	F		X					SCN1A(het, dn)
165	F							SQSTM1(het)
167	F				X			SERPIND1(het, inherited), F2(het, inherited)
168	F				X			MEFV(het, inherited)
169	M							COL4A5(hem, dn)
172	F			X				LZTR1(het, dn)
175	F	X	X				X	EARS2(2 var in trans)
179	Not determined			X				PKHD1(2 var in trans)
183	F							MFN2(het, dn)
187	F	X	X					KCND3(het, dn)

Case ID	Gender	Neurodevelopmental disorders	Seizure	Syndromic/malformative	Immune/hematological diseases	Cardiovascular disease	Metabolic/hormonal anomaly	Primary Finding overview: Gene (zygosity, inheritance)
194	F			X				PTEN(het, dn)
196	M	X				X		EXOSC9(2 var in trans)
199	M				X			STAT1(het, dn)
206	F	X		X				PTPN11(het, dn)
207	M			X				PTPN11(het, dn)
211	M						X	POLG(2 var in trans)
212	F			X				RYR1(2 var, fase unknown)
218	M				X			STAT3(het, dn)
222	F						X	B4GALT1(2 var in trans)
225	M							KMT2B(het, dn)
226	F	X		X				MECP2(het, dn), ASCL1(het, dn)
233	M				X			MEFV(het)
235	M	X		X				FBXO11(het, dn)
243	F	X	X					GNAO1(het, dn)
244	F	X						ANKRD11(het, dn)
247	M				X			TNFRSF13B(het, inherited)
248	M			X				GPC3(hem)
249	F							PSEN2(het)
263	M						X	ANO5(2 var in trans)
272	F	X	X	X				SCN2A(het, dn)
276	F	X		X				PUF60(het, dn)
280	M				X			UNC13D(2 var in trans)
282	M				X			MPL(2 var in trans)
292	F	X		X				KMT2A(het, dn)
293	M	X		X				KAT6B(het, dn)
294	F	X		X				ANKRD11(het, dn)
300	F	X		X				HECW2(het, dn)
302	F	X	X					ENTPD1(hom)
308	F	X	X	X				ATRX(het)
309	M	X	X	X				CASR(het, inherited)
311	F	X				X		SATB2(het, inherited)
315	M			X				NFIA(het, dn)
316	M							FGA(het)
318	M		X					KCNT1(het, dn)
320	M				X			PRF1(hom)
321	M							MYO7A(2 var in trans)
323	F	X						NPC1(hom)
325	F							MSH2(het)
328	F	X		X				KMT2D(het, dn)

Case ID	Gender	Neurodevelopmental disorders	Seizure	Syndromic/malformative	Immune/hematological diseases	Cardiovascular disease	Metabolic/hormonal anomaly	Primary Finding overview: Gene (zygosity, inheritance)
330	M						X	ABCB4(2 var in trans)
331	M				X			FLG(het)
332	F							VHL(het)
334	M	X	X	X				AHDC1(het, dn)
336	M	X		X				DYRK1A(het, dn)
342	M	X	X					SCN8A(het, dn)
344	F		X				X	PRRT2(het, inherited)
346	F	X	X	X				MBTPS2(het, inherited)
349	M					X		TMEM127(het)
350	M	X		X				SCN2A(het, dn)
352	F	X		X				POU3F3(het, dn)
354	Not determined			X				FGFR3(het, dn)
356	F			X				PTEN(het)
359	M	X	X					GLB1(2 var in trans)
362	M					X		MYBPC3(het)
364	F				X			CARD14(het, inherited)
369	F							PKD1(het, dn)
371	M	X	X					HEXA(hom)
375	M	X	X	X				PTEN(het)
376	M	X		X				ANKRD11(het, dn)
380	M	X						NPC1(2 var in trans)
381	F	X	X	X		X	X	PLCB4(het, dn)
382	F	X						SCN8A(het, dn), SMARCA4(het, dn)
386	F			X				EBP(het, dn)
389	F	X		X	X	X	X	FOXP1(het, dn)
391	M				X			IKBKG(hem)
397	M	X						EHMT1(het, dn)
401	M	X	X	X				MEF2C(het, dn)
405	M	X	X					DEAF1(het, dn)
412	M	X	X	X				NSD1(het, dn)
413	F	X		X				EDAR(het, inherited)
415	M				X			BTK(hem)
416	M	X	X	X				MECP2(hem, dn)
420	M	X	X					CACNA1A(het, dn), TCF12(het, inherited)
422	M				X			CTLA4(het, inherited)
424	M						X	MPV17(hom)
425	F	X						MTO1(2 var in trans)
426	M	X		X				COL2A1(het, dn)

Case ID	Gender	Neurodevelopmental disorders	Seizure	Syndromic/malformative	Immune/hematological diseases	Cardiovascular disease	Metabolic/hormonal anomaly	Primary Finding overview: Gene (zygosity, inheritance)
427	Not determined			X				PTPN11(het, dn)
437	M							KIDINS220(het)
439	M			X				COL2A1(het, dn)
443	Not determined			X				HRAS(het, dn)
444	F	X		X				KMT2A(het, dn)
446	M	X						POLG(2 var in cis)
448	F	X		X		X		DDX3X(het, dn)
451	M				X			TNFRSF13B(2 var, fase unknown)
452	F	X	X	X	X	X		MAP2K2(het, dn)
454	M	X		X				DEAF1(hom)
457	F	X						EBF3(het, dn)
460	M			X				ASNS(2 var, fase unknown)
462	F	X		X		X		NAA10(het, dn)
464	M				X			TNFRSF13B(het, inherited)
468	M			X		X		RIT1(het, dn)
478	F							TGIF1(het)
481	F	X		X				ASXL3(het, dn)
482	M	X						ECHS1(2 var in trans)
489	F	X					X	NPC1(2 var in trans)
490	M	X	X	X				THOC2(hem, inherited)
493	M			X		X	X	MYRF(het, dn)
