# Supplementary material for: Exome sequencing and targeted gene panels: a simulated comparison of diagnostic yield using data from 158 patients with rare diseases
Source: Genet Mol Biol. 2021 Sep 29;44(4):20210061. doi: 10.1590/1678-4685-GMB-2021-0061 (PMC8485181; doi:10.1590/1678-4685-GMB-2021-0061)
Supplement: Table S2 ‒ [file 1415-4757-GMB-44-4-e20210061-s2.pdf]

Genetics and Molecular Biology

DOI: https://doi.org/10.1590/1678-4685-GMB-2021-0061

Supplementary Material to "Exome sequencing and targeted gene panels: a simulated comparison of diagnostic yield using data from 158 patients with rare diseases"

Table S2- Neurodevelopmental disorders panels.

Case ID	Gender	Age	Neurodevelopmental disorders	Primary Finding overview: Gene (zygosity, inheritance)	Lab A	Lab B	Lab C	Lab D1	Lab D2	Lab E	Lab F1	Lab F2	Lab G	Lab H1	Lab H2
334	M	1.75	X	AHDC1(het, dn)	No	No	No	No	Yes	No	No	No	No	Yes	Yes
244	F	10.583	X	ANKRD11(het, dn)	Yes	No	Yes	No	Yes	Yes	Yes	Yes	No	Yes	Yes
294	F	9.833	X	ANKRD11(het, dn)	Yes	No	Yes	No	Yes	Yes	Yes	Yes	No	Yes	Yes
376	M	1.833	X	ANKRD11(het, dn)	Yes	No	Yes	No	Yes	Yes	Yes	Yes	No	Yes	Yes
27	F	2.833	X	ARID1B(het, dn)	No	No	Yes	No	Yes	Yes	Yes	Yes	No	Yes	Yes
481	F	0.833	X	ASXL3(het, dn)	No	No	No	No	Yes	No	Yes	Yes	No	Yes	Yes
148	M	7.083	X	ATP1A3(het, dn)	No	No	No	No	No	Yes	Yes	Yes	No	No	Yes
308	F	0.916	X	ATRX(het)	Yes	No	No	No	Yes	No	Yes	Yes	Yes	Yes	Yes
41	F	29	X	BLM(hom)	No	No	No	No	Yes	No	No	No	No	No	Yes
420	M	4.166	X	CACNA1A(het, dn), TCF12(het, inherited)	No	No	No	No	Yes/No	No	Yes/No	No	No/No	Yes/No	Yes/Yes
309	M	4.75	X	CASR(het, inherited)	No	No	No	No	No	No	No	Yes	No	No	No

Case ID	Gender	Age	Neurodevelopmental disorders	Primary Finding overview: Gene (zygosity, inheritance)	Lab A	Lab B	Lab C	Lab D1	Lab D2	Lab E	Lab F1	Lab F2	Lab G	Lab H1	Lab H2
426	M	5.416	X	COL2A1(het, dn)	No	No	No	No	No	No	No	Yes	No	No	No
448	F	27	X	DDX3X(het, dn)	No	No	Yes	No	Yes	No	Yes	Yes	No	Yes	Yes
99	M	7	X	DEAF1(het, dn)	No	No	No	No	Yes	Yes	No	No	No	No	Yes
405	M	14.333	X	DEAF1(het, dn)	No	No	No	No	Yes	Yes	No	No	No	No	Yes
454	M	2.916	X	DEAF1(hom)	No	No	No	No	Yes	Yes	No	No	No	No	Yes
38	M	16.333	X	DYNC1H1(het, dn)	No	No	No	No	Yes	No	Yes	Yes	No	Yes	Yes
336	M	6.25	X	DYRK1A(het, dn)	No	No	Yes	No	Yes	Yes	Yes	Yes	Yes	Yes	Yes
175	F	2.916	X	EARS2(2 var in trans)	No	No	No	No	No	No	No	Yes	No	No	Yes
457	F	3	X	EBF3(het, dn)	No	No	No	No	No	No	No	No	No	No	Yes
482	M	28	X	ECHS1(2 var in trans)	No	No	No	No	No	No	No	Yes	No	No	Yes
413	F	4.333	X	EDAR(het, inherited)	No	No	No	No		No	No	No	No	No	No
397	M	7.916	X	EHMT1(het, dn)	Yes	No	Yes	No	Yes	Yes	Yes	Yes	Yes	Yes	Yes
302	F	14.25	X	ENTPD1(hom)	No	No	No	No	No	No	No	Yes	No	No	Yes
196	M	0.166	X	EXOSC9(2 var in trans)	No	No	No	No	No	No	No	No	No	No	No
37	M	18.166	X	FAM111A(het, dn)	No	No	No	No	Yes	No	No	No	No	No	Yes
235	M	3.833	X	FBXO11(het, dn)	No	No	No	No	Yes	No	No	No	No	No	Yes
389	F	13.833	X	FOXP1(het, dn)	Yes	No	Yes	No	Yes	Yes	Yes	Yes	No	Yes	Yes
359	M	2.333	X	GLB1(2 var in trans)	No	No	No	No	Yes	No	No	Yes	No	No	Yes
84	M	0.416	X	GNAO1(het, dn)	No	No	No	No	Yes	No	Yes	Yes	No	Yes	Yes
243	F	7.416	X	GNAO1(het, dn)	No	No	No	No	Yes	No	Yes	Yes	No	Yes	Yes
124	F	2.166	X	GRIN2A(het, dn)	No	No	No	No	Yes	No	Yes	Yes	No	No	Yes
300	F	2.083	X	HECW2(het, dn)	No	No	No	No	No	No	No	No	No	No	Yes
371	M	6.333	X	HEXA(hom)	No	No	No	No	Yes	No	No	Yes	No	No	Yes

Case ID	Gender	Age	Neurodevelopmental disorders	Primary Finding overview: Gene (zygosity, inheritance)	Lab A	Lab B	Lab C	Lab D1	Lab D2	Lab E	Lab F1	Lab F2	Lab G	Lab H1	Lab H2
139	M	1	X	ITGA8(2 var in trans), PHF8(hem, inherited)	No	No	No	No	No	No	No/Yes	No/Yes	No	No	No
82	F	4.75	X	ITPR1(het, dn)	No	No	No	No	Yes	No	Yes	Yes	No	Yes	Yes
293	M	11.5	X	KAT6B(het, dn)	No	No	No	No	Yes	No	Yes	Yes	No	Yes	Yes
187	F	11.833	X	KCND3(het, dn)	No	No	No	No		No	Yes	Yes	No	No	Yes
292	F	1.666	X	KMT2A(het, dn)	No	No	Yes	No	Yes	No	Yes	Yes	No	Yes	Yes
444	F	24	X	KMT2A(het, dn)	No	No	Yes	No	Yes	No	Yes	Yes	No	Yes	Yes
328	F	15.583	X	KMT2D(het, dn)	No	No	No	No	Yes	No	Yes	Yes	No	Yes	Yes
452	F	0.833	X	MAP2K2(het, dn)	No	No	No	No	Yes	No	No	No	No	No	Yes
346	F	1.083	X	MBTPS2(het, inherited)	No	No	No	No	Yes	No	Yes	Yes	No	No	Yes
416	M	0.583	X	MECP2(hem, dn)	Yes	No	Yes	Yes	Yes	Yes	Yes	Yes	Yes	Yes	Yes
121	M	2.166	X	MECP2(hem, inherited)	Yes	No	Yes	Yes	Yes	Yes	Yes	Yes	No	Yes	Yes
22	F	14.416	X	MECP2(het, dn)	Yes	No	Yes	Yes	Yes	Yes		Yes	No	Yes	Yes
78	F	1.666	X	MECP2(het, dn)	Yes	No	Yes	Yes	Yes	Yes	Yes	Yes	No	Yes	Yes
136	F	7.833	X	MECP2(het, dn)	Yes	No	Yes	Yes	Yes	Yes	Yes	Yes	No	Yes	Yes
140	F	6.583	X	MECP2(het, dn)	Yes	No	Yes	Yes	Yes	Yes	Yes	Yes	No	Yes	Yes
226	F	2.083	X	MECP2(het, dn), ASCL1(het, dn)	Yes/No	No	Yes/No	Yes/No	Yes/No	Yes/No	Yes/Yes	Yes/Yes	Yes/No	Yes/No	Yes/Yes
401	M	5.25	X	MEF2C(het, dn)	Yes	No	No	No	Yes	Yes	Yes	Yes	Yes	Yes	Yes
425	F	37	X	MTO1(2 var in trans)	No	No	No	No	No	No	No	Yes	No	No	Yes
133	M	2.916	X	MYO7A(2 var in trans)	No	No	No	No	No	No	No	Yes	No	No	No
462	F	1.833	X	NAA10(het, dn)	No	No	No	No	Yes	No	Yes	Yes	No	No	No
					40	54	37	47	12	35	21	12	47	26	7
