# Supplementary material for: Exome sequencing and targeted gene panels: a simulated comparison of diagnostic yield using data from 158 patients with rare diseases
Source: Genet Mol Biol. 2021 Sep 29;44(4):20210061. doi: 10.1590/1678-4685-GMB-2021-0061 (PMC8485181; doi:10.1590/1678-4685-GMB-2021-0061)
Supplement: Table S3 - [file 1415-4757-GMB-44-4-e20210061-s3.pdf]

Genetics and Molecular Biology
DOI: https://doi.org/10.1590/1678-4685-GMB-2021-0061

Supplementary Material to "Exome sequencing and targeted gene panels: a simulated comparison of diagnostic yield using data from 158 patients with rare diseases"

Table S3 – Seizure panels.

Case ID	Gender	Age	Seizure	Primary Finding overview: Gene (zygosity, inheritance)	Primary Finding: [illegible]	Lab A1	Lab A2	Lab B	Lab C	Lab D	Lab E1	Lab E2	Lab F	Lab G	Lab H1	Lab H2	Lab H3
6	M	0.583	X	NFIX(het, dn)	AD	No	No	No	No	No	No	No	No	No	No	No	Yes
18	F	7.25	X	SLC13A5(hom)	AR	No	Yes	No	Yes	Yes	Yes	No	Yes	Yes	Yes	Yes	Yes
22	F	14.42	X	MECP2(het, dn)	X-linked	No	Yes	Yes	Yes	Yes	Yes	Yes	Yes	Yes	Yes	Yes	Yes
33	M	5.666	X	UPF3B(hem, inherited)	X-linked	No	No	No	No	No	No	No	No	No	No	No	Yes
44	M	4.25	X	STXBP1(het, dn)	AD	No	Yes	Yes	Yes	Yes	Yes	No	Yes	Yes	Yes	Yes	Yes
84	M	0.416	X	GNAO1(het, dn)	AD	Yes	No	No	Yes	Yes	Yes	No	Yes	Yes	Yes	Yes	Yes
87	F	3.333	X	SLC2A1(het, dn)	AD	Yes	Yes	Yes	Yes	Yes	Yes	No	Yes	Yes	Yes	Yes	Yes
99	M	7	X	DEAF1(het, dn)	AD	No	No	No	Yes	Yes	Yes	No	No	No	No	No	Yes
120	F	27	X	POLG(2 var in cis)	AD	Yes	Yes	Yes	Yes	Yes	Yes	No	Yes	Yes	Yes	Yes	Yes
121	M	2.166	X	MECP2(hem, inherited)	X-linked	No	Yes	Yes	Yes	Yes	Yes	Yes	Yes	Yes	Yes	Yes	Yes
124	F	2.166	X	GRIN2A(het, dn)	AD	Yes	Yes	Yes	Yes	Yes	Yes	No	Yes	Yes	Yes	Yes	Yes
148	M	7.083	X	ATP1A3(het, dn)	AD	No	No	No	No	Yes	Yes	No	No	Yes	Yes	Yes	Yes
161	F	7.833	X	SCN1A(het, dn)	AD	Yes	Yes	Yes	Yes	Yes	Yes	No	Yes	Yes	Yes	Yes	Yes

Case ID	Gender	Age	Seizure	Primary Finding overview: Gene (zygosity, inheritance)	Primary Finding: [illegible]	Lab A1	Lab A2	Lab B	Lab C	Lab D	Lab E1	Lab E2	Lab F	Lab G	Lab H1	Lab H2	Lab H3
175	F	2.916	X	EARS2(2 var in trans)	AR	No	No	No	No	Yes	Yes	No	Yes	No	No	No	Yes
187	F	11.83	X	KCND3(het, dn)	AD	No	No	No	No	No	No	No	No	No	No	No	Yes
243	F	7.416	X	GNAO1(het, dn)	AD	Yes	No	No	Yes	Yes	Yes	No	Yes	Yes	Yes	Yes	Yes
272	F	0.25	X	SCN2A(het, dn)	AD	No	Yes	Yes	Yes	Yes	Yes	No	Yes	Yes	Yes	Yes	Yes
302	F	14.25	X	ENTPD1(hom)	AR	No	No	No	No	No	No	No	No	No	No	No	No
308	F	0.916	X	ATRX(het)	X-linked	Yes	No	No	No	Yes	Yes	No	No	Yes	Yes	Yes	Yes
309	M	4.75	X	CASR(het, inherited)	AD	Yes	No	No	Yes	No	Yes	No	No	Yes(additional)	No	No	No
318	M	0.083	X	KCNT1(het, dn)	AD	Yes	Yes	No	Yes	Yes	Yes	No	Yes	Yes	Yes	Yes	Yes
334	M	1.75	X	AHDC1(het, dn)	AD	No	No	No	No	No	No	No	No	No	No	No	No
342	M	0.5	X	SCN8A(het, dn)	AD	No	Yes	Yes	Yes	Yes	Yes	Yes	Yes	Yes	Yes	Yes	Yes
344	F	0.416	X	PRRT2(het, inherited)	AD	No	Yes	Yes	Yes	Yes	Yes	No	Yes	Yes	Yes	Yes	Yes
346	F	1.083	X	MBTPS2(het, inherited)	X-linked	No	No	No	No	No	No	No	No	No	No	No	Yes
359	M	2.333	X	GLB1(2 var in trans)	AR	Yes	No	No	No	Yes	Yes	No	Yes	No	No	No	Yes
371	M	6.333	X	HEXA(hom)	AR	Yes	No	No	Yes	Yes	Yes	No	No	No	No	No	Yes
375	M	1.166	X	PTEN(het)	AD	No	No	No	No	Yes	Yes	No	No	Yes additional	No	No	Yes
381	F	2.25	X	PLCB4(het, dn)	AD	No	No	No	No	No	No	No	No	No	No	No	No
401	M	5.25	X	MEF2C(het, dn)	AD	Yes	Yes	Yes	Yes	Yes	Yes	Yes	Yes	Yes	Yes	Yes	Yes
405	M	14.33	X	DEAF1(het, dn)	AD	No	No	No	Yes	Yes	Yes	No	No	No	No	No	Yes
412	M	1.583	X	NSD1(het, dn)	AD	Yes	No	No	No	Yes	No	No	No	No	No	No	Yes
416	M	0.583	X	MECP2(hem, dn)	X-linked	No	Yes	Yes	Yes	Yes	Yes	Yes	Yes	Yes	Yes	Yes	Yes
420	M	4.166	X	CACNA1A(het, dn), TCF12(het, inherited)	AD and AD	Yes/No	No/No	No/No	Yes/No	Yes	Yes/No	Yes/No	Yes/No	Yes/No	Yes/No	Yes/No	Yes/No

Case ID	Gender	Age	Seizure	Primary Finding overview: Gene (zygosity, inheritance)	Primary Finding: [illegible]	Lab A1	Lab A2	Lab B	Lab C	Lab D	Lab E1	Lab E2	Lab F	Lab G	Lab H1	Lab H2	Lab H3
452	F	0.833	X	MAP2K2(het, dn)	AD	No	No	No	No	Yes	Yes	No	No	No	No	No	Yes
490	M	4.333	X	THOC2(hem, inherited)	X-linked	No	No	No	No	No	No	No	No	No	No	No	No
						22	21	23	15	9	9	30	17	15	17	17	5
