# Supplementary material for: Exome sequencing and targeted gene panels: a simulated comparison of diagnostic yield using data from 158 patients with rare diseases
Source: Genet Mol Biol. 2021 Sep 29;44(4):20210061. doi: 10.1590/1678-4685-GMB-2021-0061 (PMC8485181; doi:10.1590/1678-4685-GMB-2021-0061)
Supplement: Table S4 ‒ [file 1415-4757-GMB-44-4-e20210061-s4.pdf]

Genetics and Molecular Biology
DOI: https://doi.org/10.1590/1678-4685-GMB-2021-0061

Supplementary Material to "Exome sequencing and targeted gene panels: a simulated comparison of diagnostic yield using data from 158 patients with rare diseases"

Table S4 - Syndromic/malformative panels.

Case ID	Gender	Age	Syndromic/malformative	Primary Finding overview: Gene (zygosity, inheritance)	Primary Finding: Inheritance	Lab A	Lab B	Lab C	Lab D	Lab E	Lab F	Lab G	Lab H
4	M	2	X	SLC52A2(hom)	AR	No	No	No	No	No	No	No	No
6	M	0.583	X	NFIX(het, dn)	AD	No	Yes	Yes	Yes	No	No	No	No
8	M	6.25	X	CSNK2A1(het, dn)	AD	No	No	No	No	No	No	No	No
22	F	14.416	X	MECP2(het, dn)	X-linked	No	No	No	No	No	No	No	No
26	M	2	X	PQBP1(hem, inherited)	X-linked	No	No	No	Yes	No	Yes	No	No
27	F	2.833	X	ARID1B(het, dn)	AD	No	Yes	Yes	No	No	Yes	No	No
28	F	3	X	ZC4H2(het)	X-linked	No	No	No	No	No	No	No	No
33	M	5.666	X	UPF3B(hem, inherited)	X-linked	No	No	No	No	No	No	No	No
37	M	18.166	X	FAM111A(het, dn)	AD	No	No	No	No	No	No	No	No
41	F	29	X	BLM(hom)	AR	No	Yes	Yes	Yes	No	No	No	No
44	M	4.25	X	STXBP1(het, dn)	AD	No	No	No	No	No	No	No	No
84	M	0.416	X	GNAO1(het, dn)	AD	No	No	No	No	No	No	No	No
108	M	0.666	X	ASXL1(het, dn)	AD	No	Yes	Yes	Yes	No	Yes	No	No
110	F	4.75	X	PHF6(het, dn)	X-linked	No	Yes	Yes	No	No	Yes	No	No
119	M	44	X	TGFBR2(het, inherited)	AD	No	Yes	Yes	Yes	No	Yes	No	No

Case ID	Gender	Age	Syndromic/malformative	Primary Finding overview: Gene (zygosity, inheritance)	Primary Finding: Inheritance	Lab A	Lab B	Lab C	Lab D	Lab E	Lab F	Lab G	Lab H
121	M	2.166	X	MECP2(hem, inherited)	X-linked	No	No	No	No	No	No	No	No
124	F	2.166	X	GRIN2A(het, dn)	AD	No	No	No	No	No	No	No	No
127	F	10.416	X	LZTR1(het, dn)	AD	No	No	Yes	Yes	No	Yes	No	No
129	F	39	X	FBN1(het, dn)	AD	No	No	Yes	No	No	Yes	No	No
136	F	7.833	X	MECP2(het, dn)	X-linked	No	No	No	No	No	No	No	No
139	M	1	X	ITGA8(2 var in trans), PHF8(hem, inherited)	AR and X-linked	No	No/Yes	No	No/Yes	No	No/No	No	No
140	F	6.583	X	MECP2(het, dn)	X-linked	No	No	No	No	No	No	No	No
155	F	10.25	X	COL6A1(het, dn)	AD	No	No	No	No	No	Yes	No	No
172	F	0.083	X	LZTR1(het, dn)	AD	No	No	Yes	Yes	No	Yes	No	No
179	Not determined	Prenatal sample	X	PKHD1(2 var in trans)	AR	No	No	No	Yes	No	Yes	No	No
194	F	20.75	X	PTEN(het, dn)	AD	No	No	Yes	No	No	No	No	No
206	F	1.5	X	PTPN11(het, dn)	AD	No	Yes	Yes	Yes	No	Yes	No	No
207	M	0.066	X	PTPN11(het, dn)	AD	No	Yes	Yes	Yes	No	Yes	No	No
212	F	4.833	X	RYR1(2 var, fase unknown)	AR	No	No	No	No	No	Yes	No	No
226	F	2.083	X	MECP2(het, dn), ASCL1(het, dn)	AD and X-linked	No	No/No	No/No	No	No	No/No	No	No
235	M	3.833	X	FBXO11(het, dn)	AD	No	No	No	No	No	No	No	No
248	M	0.052	X	GPC3(hem)	X-linked	No	Yes	Yes	Yes	No	No	No	No
272	F	0.25	X	SCN2A(het, dn)	AD	No	No	No	No	No	No	No	No
276	F	7.583	X	PUF60(het, dn)	AD	No	No	No	Yes	No	No	No	No
292	F	1.666	X	KMT2A(het, dn)	AD	No	Yes	Yes	No	No	Yes	No	No
293	M	11.5	X	KAT6B(het, dn)	AD	No	No	Yes	No	No	Yes	No	No
294	F	9.833	X	ANKRD11(het, dn)	AD	No	Yes	Yes	Yes	No	Yes	No	No
300	F	2.083	X	HECW2(het, dn)	AD	No	No	No	No	No	No	No	No
308	F	0.916	X	ATRX(het)	X-linked	No	No	Yes	Yes	No	Yes	No	No
309	M	4.75	X	CASR(het, inherited)	AD	No	No	No	No	No	Yes	No	No
315	M	3.916	X	NFIA(het, dn)	AD	No	No	No	No	No	No	No	No
328	F	15.583	X	KMT2D(het, dn)	AD	No	Yes	Yes	Yes	No	Yes	No	No

Case ID	Gender	Age	Syndromic/malformative	Primary Finding overview: Gene (zygosity, inheritance)	Primary Finding; Inheritance	Lab A	Lab B	Lab C	Lab D	Lab E	Lab F	Lab G	Lab H
334	M	1.75	X	AHDC1(het, dn)	AD	No	No	No	No	No	No	No	No
336	M	6.25	X	DYRK1A(het, dn)	AD	No	No	No	Yes	No	Yes	No	No
346	F	1.083	X	MBTPS2(het, inherited)	X-linked	No	Yes	No	Yes	No	Yes	No	No
350	M	6.25	X	SCN2A(het, dn)	AD	No	No	No	No	No	No	No	No
352	F	28	X	POU3F3(het, dn)	AD	No	No	No	No	No	No	No	No
354	Not determined	Prenatal sample	X	FGFR3(het, dn)	AD	No	Yes	No	Yes	No	Yes	No	No
356	F	54	X	PTEN(het)	AD	No	No	Yes	No	No	No	No	No
375	M	1.166	X	PTEN(het)	AD	No	No	Yes	No	No	No	No	No
376	M	1.833	X	ANKRD11(het, dn)	AD	No	Yes	Yes	Yes	No	Yes	No	No
381	F	2.25	X	PLCB4(het, dn)	AD	No	Yes	No	Yes	No	No	No	No
386	F	0.583	X	EBP(het, dn)	X-linked	No	Yes	No	Yes	No	Yes	No	No
389	F	13.833	X	FOXP1(het, dn)	AD	No	No	No	No	No	No	No	No
401	M	5.25	X	MEF2C(het, dn)	AD	No	No	No	No	No	No	No	No
412	M	1.583	X	NSD1(het, dn)	AD	No	Yes	Yes	Yes	No	No	No	No
413	F	4.333	X	EDAR(het, inherited)	AD	No	No	No	No	No	No	No	No
416	M	0.583	X	MECP2(hem, dn)	X-linked	No	No	No	No	No	No	No	No
426	M	5.416	X	COL2A1(het, dn)	AD	No	Yes	No	Yes	No	Yes	No	No
427	Not determined	Prenatal sample	X	PTPN11(het, dn)	AD	No	Yes	Yes	Yes	No	Yes	No	No
439	M	0.5	X	COL2A1(het, dn)	AD	No	Yes	No	Yes	No	Yes	No	No
443	Not determined	Prenatal sample	X	HRAS(het, dn)	AD	No	Yes	Yes	Yes	No	Yes	No	No
444	F	24	X	KMT2A(het, dn)	AD	No	Yes	Yes	No	No	Yes	No	No
448	F	27	X	DDX3X(het, dn)	AD	No	No	No	No	No	No	No	No
452	F	0.833	X	MAP2K2(het, dn)	AD	No	Yes	Yes	Yes	No	Yes	No	No
454	M	2.916	X	DEAF1(hom)	AR	No	No	No	No	No	No	No	No
460	M	0.25	X	ASNS(2 var, fase unknown)	AR	No	No	No	No	No	No	No	No

Case ID	Gender	Age	Syndromic/malformative	Primary Finding overview: Gene (zygosity, inheritance)	Primary Finding: Inheritance	Lab A	Lab B	Lab C	Lab D	Lab E	Lab F	Lab G	Lab H
462	F	1.833	X	NAA10(het, dn)	X-linked	No	No	No	No	No	Yes	No	No
468	M	0.083	X	RIT1(het, dn)	AD	No	Yes	Yes	Yes	No	No	No	No
481	F	0.833	X	ASXL3(het, dn)	AD	No	No	No	No	No	Yes	No	No
490	M	4.333	X	THOC2(hem, inherited)	X-linked	No	No	No	No	No	No	No	No
493	M	1.5	X	MYRF(het, dn)	AD	No	No	No	No	No	No	No	No
						72	45	44	43	72	38	72	72
