# Supplementary material for: Exome sequencing and targeted gene panels: a simulated comparison of diagnostic yield using data from 158 patients with rare diseases
Source: Genet Mol Biol. 2021 Sep 29;44(4):20210061. doi: 10.1590/1678-4685-GMB-2021-0061 (PMC8485181; doi:10.1590/1678-4685-GMB-2021-0061)
Supplement: Table S5 ‒ [file 1415-4757-GMB-44-4-e20210061-s5.pdf]

Genetics and Molecular Biology
DOI: https://doi.org/10.1590/1678-4685-GMB-2021-0061

Supplementary Material to "Exome sequencing and targeted gene panels: a simulated comparison of diagnostic yield using data from 158 patients with rare diseases"

Table S5 - Immune/hematological diseases panels.

Case ID	Gender	Age	Immune/hematological diseases	Primary Finding overview: Gene (zygosity, inheritance)	Primary Finding: Inheritance	Lab A1	Lab A2	Lab B	Lab C1	Lab C2	Lab D	Lab E	Lab F	Lab G	Lab H
167	F	24	X	SERPIND1(het, inherited), F2(het, inherited)	AD and AD	No	No	No	No	No	No	No	No	No	No
168	F	40	X	MEFV(het, inherited)	AD	No	No	Yes	Yes	No	No	Yes	Yes	Yes	Yes
199	M	10.166	X	STAT1(het, dn)	AD	No	No	Yes	Yes	Yes	No	Yes	Yes	Yes	Yes
218	M	2.75	X	STAT3(het, dn)	AD	Yes	No	Yes	Yes	No	No	Yes	Yes	Yes	Yes
233	M	29	X	MEFV(het)	AD	No	No	Yes	Yes	No	No	Yes	Yes	Yes	Yes
247	M	3.333	X	TNFRSF13B(het, inherited)	AD	No	No	Yes	Yes	No	No	Yes	Yes	Yes	Yes
280	M	2.416	X	UNC13D(2 var in trans)	AR	No	No	Yes	Yes	Yes	No	Yes	Yes	Yes	Yes
282	M	2.5	X	MPL(2 var in trans)	AR	No	No	No	Yes	No	No	Yes	No	No	No
320	M	12.333	X	PRF1(hom)	AR	No	No	Yes	Yes	Yes	No	Yes	Yes	Yes	No
331	M	44	X	FLG(het)	AD	No	No	No	No	No	No	No	No	No	No
364	F	37	X	CARD14(het, inherited)	AD	No	No	Yes	No	No	No	Yes	No	Yes	No
389	F	13.833	X	FOXP1(het, dn)	AD	No	No	No	No	No	No	No	No	No	No
391	M	1.25	X	IKBKG(hem)	X-linked	Yes	No	Yes	Yes	No	No	Yes	Yes	No	Yes
415	M	1.583	X	BTK(hem)	X-linked	No	No	Yes	Yes	Yes	No	Yes	Yes	Yes	Yes
422	M	13.416	X	CTLA4(het, inherited)	AD	No	No	Yes	Yes	No	No	Yes	Yes	Yes	Yes
451	M	46	X	TNFRSF13B(2 var, fase unknown)	AR	No	No	Yes	Yes	No	No	Yes	Yes	Yes	Yes
452	F	0.833	X	MAP2K2(het, dn)	AD	No	No	No	No	No	No	No	No	No	No

Case ID	Gender	Age	Immune/hematological diseases	Primary Finding overview: Gene (zygosity, inheritance)	Primary Finding: Inheritance	Lab A1	Lab A2	Lab B	Lab C1	Lab C2	Lab D	Lab E	Lab F	Lab G	Lab H
464	M	2.583	X	TNFRSF13B(het, inherited)	AD	No	No	Yes	Yes	No	No	Yes	Yes	Yes	Yes
						16	18	5	5	14	18	4	6	6	7
