# Supplementary material for: Exome sequencing and targeted gene panels: a simulated comparison of diagnostic yield using data from 158 patients with rare diseases
Source: Genet Mol Biol. 2021 Sep 29;44(4):20210061. doi: 10.1590/1678-4685-GMB-2021-0061 (PMC8485181; doi:10.1590/1678-4685-GMB-2021-0061)
Supplement: Table S6 ‒ [file 1415-4757-GMB-44-4-e20210061-s6.pdf]

Genetics and Molecular Biology
DOI: https://doi.org/10.1590/1678-4685-GMB-2021-0061

Supplementary Material to "Exome sequencing and targeted gene panels: a simulated comparison of diagnostic yield using data from 158 patients with rare diseases"

Table S6 - Cardiovascular disease panels.

Case ID	Gender	Age	Cardiovascular disease	Primary Finding overview: Gene (zygosity, inheritance)	Primary Finding: Inheritance	Lab A1	Lab A2	Lab B	Lab C	Lab D	Lab E	Lab F	Lab G1	Lab G2	Lab H1	Lab H2
55	F	0.416	X	TNNC1(het, dn)	AD	Yes	Yes	No	Yes	Yes	Yes	Yes	No	Yes	Yes	Yes
108	M	0.666	X	ASXL1(het, dn)	AD	No	No	No	No	No	No	No	No	No	No	No
127	F	10.416	X	LZTR1(het, dn)	AD	No	No	No	No	No	Yes	Yes	No	No	No	No
134	M	3.583	X	NDUFV1(2 var in trans)	AR	No	No	No	Yes	No	No	No	No	No	No	No
136	F	7.833	X	MECP2(het, dn)	X-linked	No	No	No	No	No	No	No	No	No	No	No
196	M	0.166	X	EXOSC9(2 var in trans)	AR	No	No	No	No	No	No	No	No	No	No	No
311	F	7.166	X	SATB2(het, inherited)	AD	No	No	No		No	No	No	No	No	No	
349	M	39	X	TMEM127(het)	AD	No	No	No	No	No	No	No	No	No	No	No
362	M	16.416	X	MYBPC3(het)	AD	Yes	Yes	No	Yes	Yes	Yes	Yes	No	Yes	Yes	Yes
381	F	2.25	X	PLCB4(het, dn)	AD	No	No	No	No	No	No	No	No	No	No	No
389	F	13.833	X	FOXP1(het, dn)	AD	No	No	No	No	No	No	No	No	No	No	No
448	F	27	X	DDX3X(het, dn)	AD	No	No	No	No	No	No	No	No	No	No	No
452	F	0.833	X	MAP2K2(het, dn)	AD	Yes	No	Yes	Yes	No	Yes	Yes	Yes	No	Yes	Yes
462	F	1.833	X	NAA10(het, dn)	X-linked	No	No	No	No	No	No	No	No	No	No	No
468	M	0.083	X	RIT1(het, dn)	AD	No	No	Yes	Yes	No	Yes	Yes	Yes	No	Yes	Yes
493	M	1.5	X	MYRF(het, dn)	AD	No	No	No	No	No	No	No	No	No	No	No
						13	14	14	10	14	11	11	14	14	12	11
