# Supplementary material for: Exome sequencing and targeted gene panels: a simulated comparison of diagnostic yield using data from 158 patients with rare diseases
Source: Genet Mol Biol. 2021 Sep 29;44(4):20210061. doi: 10.1590/1678-4685-GMB-2021-0061 (PMC8485181; doi:10.1590/1678-4685-GMB-2021-0061)
Supplement: Table S7 - [file 1415-4757-GMB-44-4-e20210061-s7.pdf]

Genetics and Molecular Biology

DOI: https://doi.org/10.1590/1678-4685-GMB-2021-0061

Supplementary Material to "Exome sequencing and targeted gene panels: a simulated comparison of diagnostic yield using data from 158 patients with rare diseases"

Table S7 – Metabolic abnormality panels.

Case ID	Gender	Age	Metabolic/hormonal anomaly	Primary Finding overview: Gene (zygosity, inheritance)	Primary Finding: Inheritance	Lab A	Lab B	Lab C	Lab D	Lab E	Lab F1	Lab F2	Lab G1	Lab G2	Lab H1	Lab H2
77	F	10	X	TF(hom)	AR	No	No	No	No	No	No	No	No	No	No	No
82	F	4.75	X	ITPR1(het, dn)	AD	No	No	No	No	No	No	No	No	No	No	No
95	M	0.25	X	MPV17(2 var in trans)	AR	No	No	No	Yes	No	No	Yes	No	No	No	No
99	M	7	X	DEAF1(het, dn)	AD	No	No	No	No	No	No	No	No	No	No	No
120	F	27	X	POLG(2 var in cis)	AD	No	No	No	No	No	No	Yes	Yes	No	No	No
126	M	21	X	DYSF(2 var in trans), PTH1R(het, inherited)	AR and AD	No	No	No	No	No	No	No	No	No	No	No
175	F	2.916	X	EARS2(2 var in trans)	AR	No	No	No	Yes	No	No	Yes	No	No	No	No
211	M	1.166	X	POLG(2 var in trans)	AR	No	No	No	Yes	No	No	Yes	Yes	No	No	No
222	F	4.166	X	B4GALT1(2 var in trans)	AR	No	No	No	Yes	No	No	Yes	No	Yes	No	Yes
263	M	16	X	ANO5(2 var in trans)	AR	No	No	No	No	No	No	No	No	No	No	No
330	M	5.416	X	ABCB4(2 var in trans)	AR	No	No	Yes	Yes	No	Yes	Yes	No	No	No	No
344	F	0.416	X	PRRT2(het, inherited)	AD	No	No	No	No	No	No	No	No	No	No	No
381	F	2.25	X	PLCB4(het, dn)	AD	No	No	No	No	No	No	No	No	No	No	No
389	F	13.833	X	FOXP1(het, dn)	AD	No	No	No	No	No	No	No	No	No	No	No
424	M	0.75	X	MPV17(hom)	AR	No	No	No	Yes	No	No	Yes	No	No	No	No
489	F	1.333	X	NPC1(2 var in trans)	AR	Yes	Yes	Yes	Yes	Yes	Yes	Yes	Yes	No	Yes	No
493	M	1.5	X	MYRF(het, dn)	AD	No	No	No	No	No	No	No	No	No	No	No
						16	16	15	10	16	15	9	14	16	16	16
